# Cholesterol-rich diet exacerbates steatohepatitis in the STAM mouse model

**DOI:** 10.1038/s41598-026-45979-z

**Published:** 2026-04-01

**Authors:** Wenke Jonas, Pascal Gottmann, Markus Jähnert, Nora Baer, Annette Schürmann, Heike Vogel

**Affiliations:** 1 Department of Experimental Diabetology, German Institute of Human Nutrition Potsdam-Rehbruecke, Arthur-Scheunert-Allee 114-116, 14558 Nuthetal, Germany; 2 German Center for Diabetes Research, 85764 München-Neuherberg, Germany; 3 Institute of Nutritional Sciences, University of Potsdam, 14558 Nuthetal, Germany

**Keywords:** MASH, Liver fibrosis, STAM, Cholesterol, Diseases, Gastroenterology

## Abstract

Metabolic dysfunction-associated steatotic liver disease (MASLD) is a widespread chronic liver disorder spanning simple steatosis to metabolic dysfunction-associated steatohepatitis (MASH) and fibrosis. Suitable animal models are crucial for therapeutic development, yet many fail to reproduce progression to fibrosing MASH. Here, we employed the STAM model in male C57BL/6J mice to simulate MASLD and investigated whether a high-fat, high-cholesterol (HFHC) diet would intensify disease progression compared with a standard high-fat (HF) diet. HFHC feeding in the STAM model accelerated hepatic lipid accumulation, crown-like structure formation, inflammation, and fibrosis, as demonstrated by histological analyses. Transcriptomic profiling and pathway enrichment confirmed activation of lipid and cholesterol metabolism, regulation of TNF production, and inflammatory signaling. Reactome analysis further indicated alterations in extracellular matrix formation and immune system pathways, consistent with advanced fibrotic progression. In summary, integration of HFHC feeding with the STAM model produced a more severe liver phenotype that better mirrors the pathological spectrum of MASLD. This optimized model offers a valuable platform for preclinical testing of therapeutic candidates targeting MASH and fibrosis, with potential to accelerate drug development and to improve treatment strategies for MASLD.

**Supplementary Information:**

The online version contains supplementary material available at 10.1038/s41598-026-45979-z.

## Introduction

Metabolic dysfunction-associated steatotic liver disease (MASLD) encompasses a spectrum of hepatic conditions ranging from steatosis (fatty liver) to the more severe metabolic dysfunction-associated steatohepatitis (MASH), which associates with inflammation and fibrosis^1^. As the disease progresses, cirrhosis or hepatocellular carcinoma (HCC) may develop. Extensive epidemiological and clinical investigations have demonstrated a robust correlation between MASLD and a constellation of metabolic disorders, including obesity^2^, insulin resistance^3^, and type 2 diabetes (T2D)^4^. Remarkably, over 60% of individuals with T2D are diagnosed with MASLD^5^, indicating a strong interplay between these pathophysiological conditions^6^. Moreover, MASLD exerts a profound systemic impact and is associated with an increased risk of cardiovascular disease, including heart failure^7^. The mechanisms underlying this association are complex and multifaceted, including dyslipidemia, chronic inflammation, and impaired insulin signaling pathways.

Given the critical clinical impact of MASLD and its potentially harmful consequences, close monitoring and tailored interventions for individuals affected by metabolic disorders and T2D are essential. In March 2024, the US FDA approved resmetriom (Rezdiffra) as first pharmacologic therapy for adults with non-cirrhotic MASH with stage F2–F3 fibrosis. Despite this advance, lifestyle modification with emphasis on diet and exercise remains first-line therapy, and only a small number of patients undergo and benefit from bariatric surgery^8,9^. In recent years, new pharmacological treatments have emerged that promise to address this gap, including treatment with GLP1 receptor agonists (e.g., semaglutide^10,11^) or the novel dual GIP/GLP-1 receptor agonist tirzepatide^12^. However, these agents require long-term use and may not be suitable for all patients due to side effects^13^.

Because of the limited success in maintaining weight loss and metabolic improvements, a major effort is underway to test potential treatments for MASLD, particularly with regard to resolution of fibrosis. Success in this endeavour also depends on preclinical models capable of capturing the disease across its various stages. Although enormous efforts are being made to develop *in vitro* models such as spheroids/organoids or liver-on-chip to reproduce key features of MASH^14,15^, research continues to rely on *in vivo* models due to the multifactorial pathophysiology of the disease including obesity, insulin resistance, and dyslipidemia induced by a hypercaloric diet. Moreover, *in vivo* models are better suited to reflect the progressive stages of MASLD and to study the interorgan crosstalk. In recent years, a number of models have been developed that rely on genetic modifications or are based on dietary challenges, or a combination of both^16^. In contrast, the STAM model employs the chemical agent streptozotocin (STZ) to partially deplete the insulin-producing β-cells. This, in combination with a high-fat diet (HF), results in overt hyperglycemia and impaired insulin sensitivity and compares to late-stage T2D. In this model, mice exhibit rapid progression of MASLD, ranging from simple steatosis to steatohepatitis and fibrosis, and ultimately to HCC within 20 weeks^17^.

One dietary factor which seems to drive simple steatosis to MASH is cholesterol, as several studies in humans and mice have shown. In both, the level of highly toxic unesterified cholesterol levels in the liver increases as the disease progresses^18–20^. The results of animal studies support this conclusion, as a HF diet generally results in simple steatosis without necroinflammation or fibrosis. However, the combination of high-fat and high-cholesterol in diets (HFHC) has been shown to accelerate the development of the hepatic features of MASH^21–23^.

The underlying mechanisms are diverse. Accumulation of free cholesterol alters membrane composition which impairs mitochondrial function and induces endoplasmic reticulum (ER) stress promoting hepatocyte damage and lipotoxicity^24^. The precipitation of free cholesterol may lead to formation of crystals within lipid droplets of hepatocytes triggering hepatic inflammation mediated by activation of resident Kupffer cells^25,26^, similar to plaque formation in atherosclerosis^27,28^. Activated Kupffer cells release pro-inflammatory and fibrogenic cytokines^29^, such as transforming growth factor β (TGF-β) and tumor necrosis factor α (TNF-α), which amplify inflammation and activate hepatic stellate cells. This activation transforms hepatic stellate cells into collagen-producing myofibroblasts, driving fibrosis. Additionally, free cholesterol accumulation in stellate cells directly enhances their activation and extra cellular matrix (ECM) production, further promoting fibrosis in MASH progression^30^.

Overall, cholesterol is a central mediator in the progression from simple steatosis to steatohepatitis and fibrosis in MASH, exerting both systemic and localized effects on liver health. Our study highlights that dietary cholesterol markedly aggravates pathology in the STAM model.

## Results

### Impact of cholesterol-enriched diet and streptozotocin on metabolism and hepatic fat accumulation

We utilized the established murine STAM model to test whether a cholesterol-enriched high-fat diet (HFHC) is more potent than the originally described high-fat diet (HF) in inducing MASH-related features such as inflammation and fibrosis in this specific model^17,21,22^. Since our study focused on the earlier stages of MASLD rather than on hepatocellular carcinoma, the mice were studied until 16 weeks of age, the age when first nodules may develop and potentially progress to hepatocellular carcinoma^17^.

**Fig. 1:**
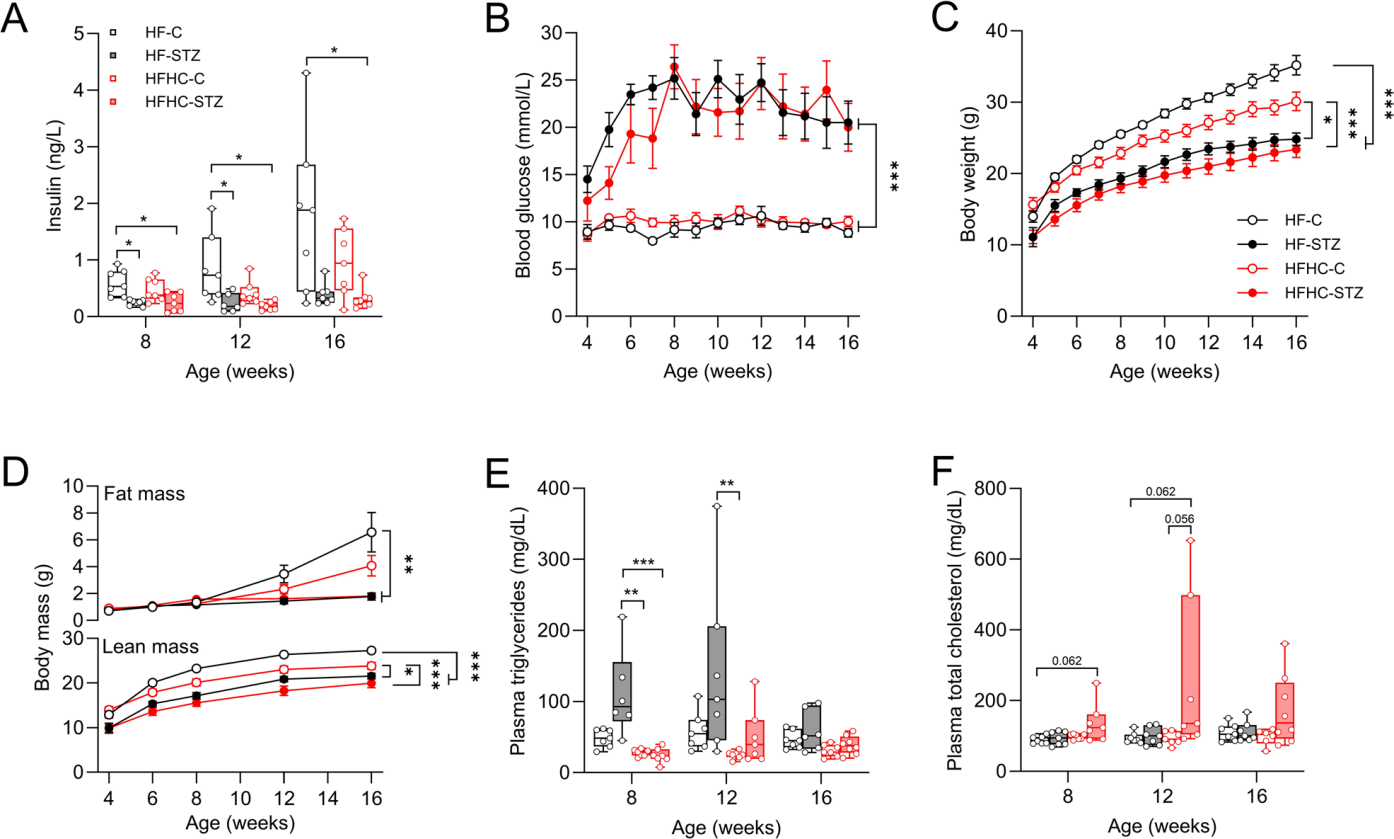
Streptozotocin treatment causes hyperglycemia. (**A**) Plasma insulin values. Development of (**B**) blood glucose and (**C**) body weight as well as (**D**) fat and lean mass during intervention. (**E**) Plasma triglycerides and (**F**) total cholesterol levels. Data are shown as mean±SEM and analyzed by non-parametric 2-way ANOVA with Tukey’s multiple comparison test. Plasma parameters are median (line), upper and lower quartile (box) and extremes (whiskers), and are analyzed by Kruskal-Wallis test for independent time points. *p < 0.05; **p < 0.01; ***p < 0.001. (*n* = 6–8/group)

As expected following STZ application, pancreatic β-cells within the Langerhans islets were partially dysfunctional, which was reflected by lower plasma insulin levels (Fig. 1A). Consequently, STZ-treated mice developed hyperglycemia (blood glucose > 16.6 mmol/L) within the first two weeks after switching to either diet, which persisted throughout the study period (Fig. 1B). In general, STZ treatment markedly attenuated body weight gain in both feeding regimes compared to controls (Fig. 1C). This, along with the low amount of body fat (Fig. 1D), are clear features of a severe diabetic state in STZ mice.

In HF-control mice (HF-C), plasma insulin levels and body fat increased with age, indicating increasing insulin resistance and compensatory hyperinsulinemia. This physiological response was attenuated in HFHC-C mice and might be explained by the lower body weight and fat mass compared to HF-C mice (Fig. S1). Strikingly, compared with HFHC mice, HF-STZ mice had significantly elevated plasma triglycerides up to 12 weeks of age, which declined by the end of the study without differences between groups (Fig. 1E). Only HFHC-STZ mice displayed elevated plasma cholesterol levels (Fig. 1F).

**Fig. 2:**
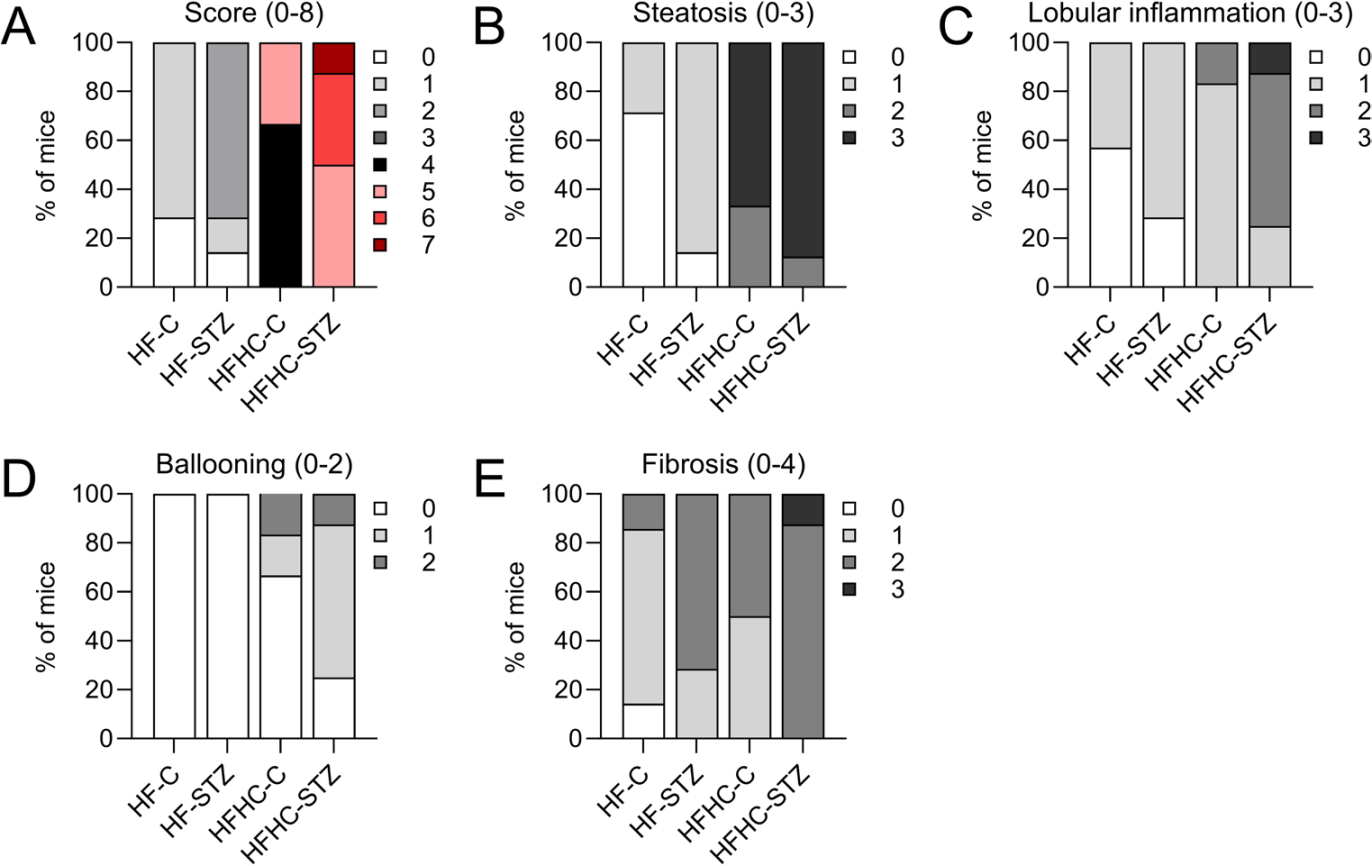
Streptozotocin treatment in combination with high-cholesterol diet increases NAFLD activity score (NAS). (**A**) Histopathological scoring by NAS calculated as sum scores for (**B**) steatosis, (**C**) lobular inflammation and (**D**) hepatocellular ballooning, and (**E**) scoring of fibrosis stage in 16-weeks old mice. (*n* = 4–7/group)

**Fig. 3:**
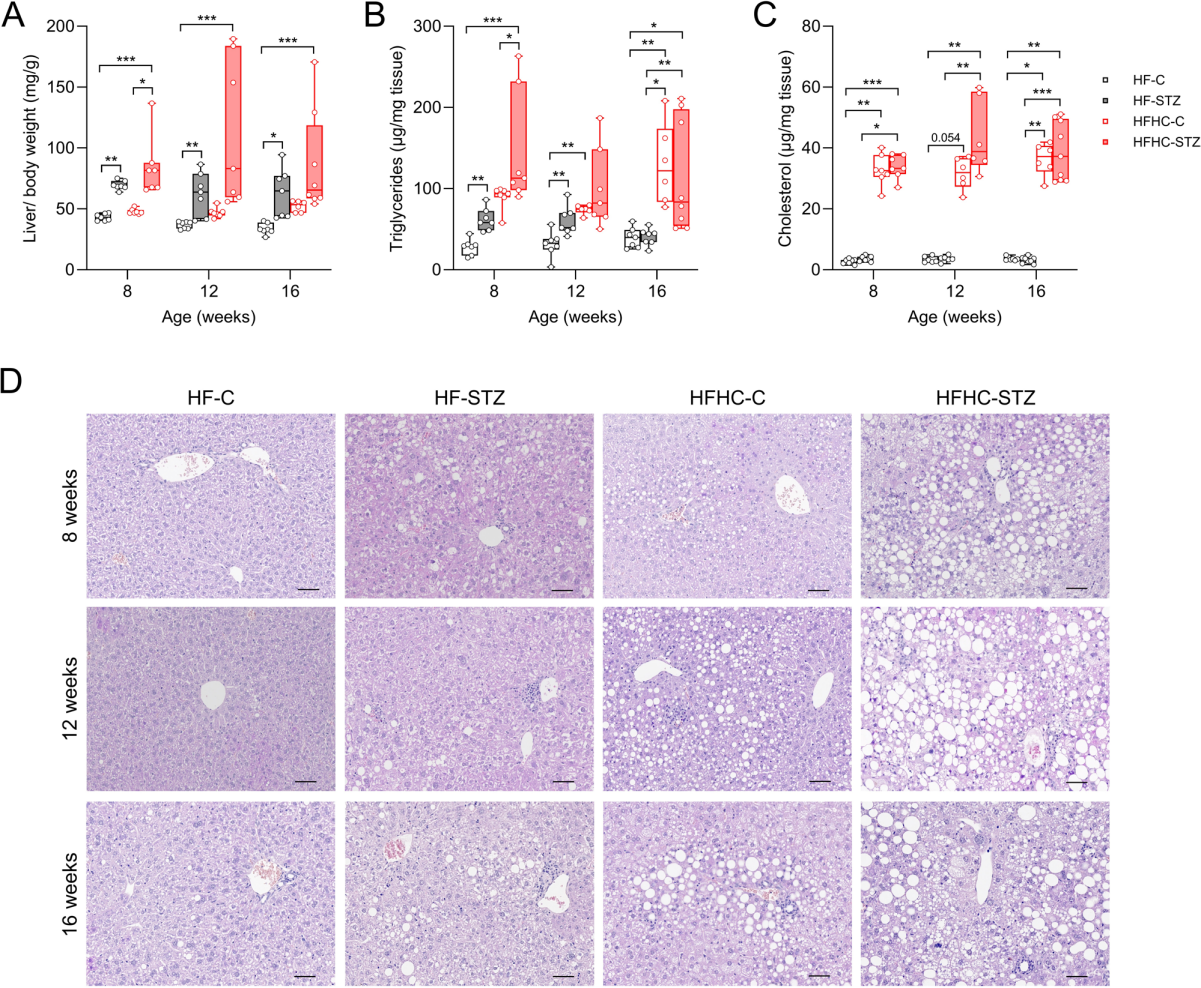
Cholesterol-enriched diet has a more severe impact on hepatic fat accumulation in STZ-treated mice. (**A**) Liver weight relative to body weight. Hepatic (**B**) triglycerides and (**C**) cholesterol levels. (**D**) Representative H&E staining of liver sections from mice at 8, 12 or 16 weeks of age (scale bars, 50 μm). Values are median (line), upper and lower quartile (box) and extremes (whiskers). Data are analyzed by Kruskal-Wallis test for independent time points. *p < 0.05; **p < 0.01; ***p < 0.001. (*n* = 6–8/group)

Subsequent analyses focused on mice aged 8 to 16 weeks, which should reflect the time to onset of steatohepatitis and progression of the disease to fibrosis and initial nodule formation. First, to assess disease severity, livers were staged using the NAFLD Activity Score (NAS). By 8 weeks, HFHC-STZ mice exhibited the highest scores, primarily driven by steatosis and lobular inflammation (Fig. S2). This pattern became more pronounced by 16 weeks (Fig. 2A-D), prompting more detailed analyses of the pathological features, namely steatosis, inflammation, and fibrosis. Both relative and absolute liver weights of STZ-treated mice was generally increased compared to control mice; however, due to variability within the HFHC group, statistical significance was reached only in HF-fed mice (Fig. 3A and Fig. S3). Consistent with relative liver weight, hepatic triglyceride content was also increased in HFHC-STZ mice at 8 and 12 weeks of age. At the end of the study, all HFHC mice had higher levels than HF mice, but STZ treatment no longer conferred an additional effect (Fig. 3B). In addition to higher hepatic triglycerides, elevated total hepatic cholesterol levels were also measured in HFHC-fed mice compared with HF-mice, reflecting the dietary cholesterol content; an effect that was not further enhanced by STZ administration (Fig. 3C). The RNAseq data indicate that the HFHC diet increases expression of genes associated with cholesterol metabolism like the ABC transporters ABCG5/G8 (*Abcg5* and *Abcg8*), which mediate the excretion of unesterified cholesterol, whereas the expression of cholesterol synthesis enzymes 3-hydroxy-3-methylglutaryl-CoA reductase (*Hmgcr*) and 3-hydroxy-3-methylglutaryl-Coenzyme A synthase 1 (*Hmgcs1*) were reduced. However, the cholesterol-metabolizing enzyme cytochrome P450 family 7a1 (*Cyp7a1*), which is pivotal in the conversion of cholesterol to bile acids, thus facilitating its excretion, was reduced, whereas *Cyp27a1* was unchanged. Finally, the enzyme acetyl-CoA acetyltransferase 2 (*Acat2*), which catalyzes the esterification of cholesterol, was reduced in HFHC-fed mice at 8 weeks but not at 12 weeks, suggesting at most a transient reduction in esterification (Fig. S4). Because free and esterified cholesterol were not quantified, we interpret potential effects on the free cholesterol pool cautiously. Nevertheless, the transcriptional profile is consistent with a potential imbalance in cholesterol handling that may favor the accumulation of lipotoxic free cholesterol in HFHC-fed livers.

Examination of H&E-stained liver sections showed an age-dependent increase in hepatic lipid accumulation in STZ mice compared to controls, independent of diet (Fig. 3D). Steatosis scores were generally highest in HFHC-fed mice and were further increased by STZ treatment (Fig. 2B and S2B, E). Steatosis may manifest in two distinct forms. Macrovesicular steatosis is marked by the existence of hepatocytes with a single droplet of large intracytoplasmic fat displacing the nucleus to the periphery, whereas microvesicular steatosis is identified by swollen hepatocytes with foamy cytoplasm and small lipid vesicles^31^. In general, especially in livers of HFHC-STZ mice, a pronounced mixture of macrovesicular and microvesicular steatosis was detected (Fig. 3D).

Collectively, these findings suggest a trend toward more severe liver pathology in HFHC-STZ mice. Consistent with this interpretation, plasma aminotransferase activities (ALT and AST), as markers of necroinflammation, were also elevated in this group (Table S1). Despite considerable inter-individual variability, the overall pattern supports increased liver damage in HFHC-STZ mice, resembling key features of human MASH^32^.

### HFHC-STZ mice develop severe hepatic inflammation and hepatic fibrosis

**Fig. 4:**
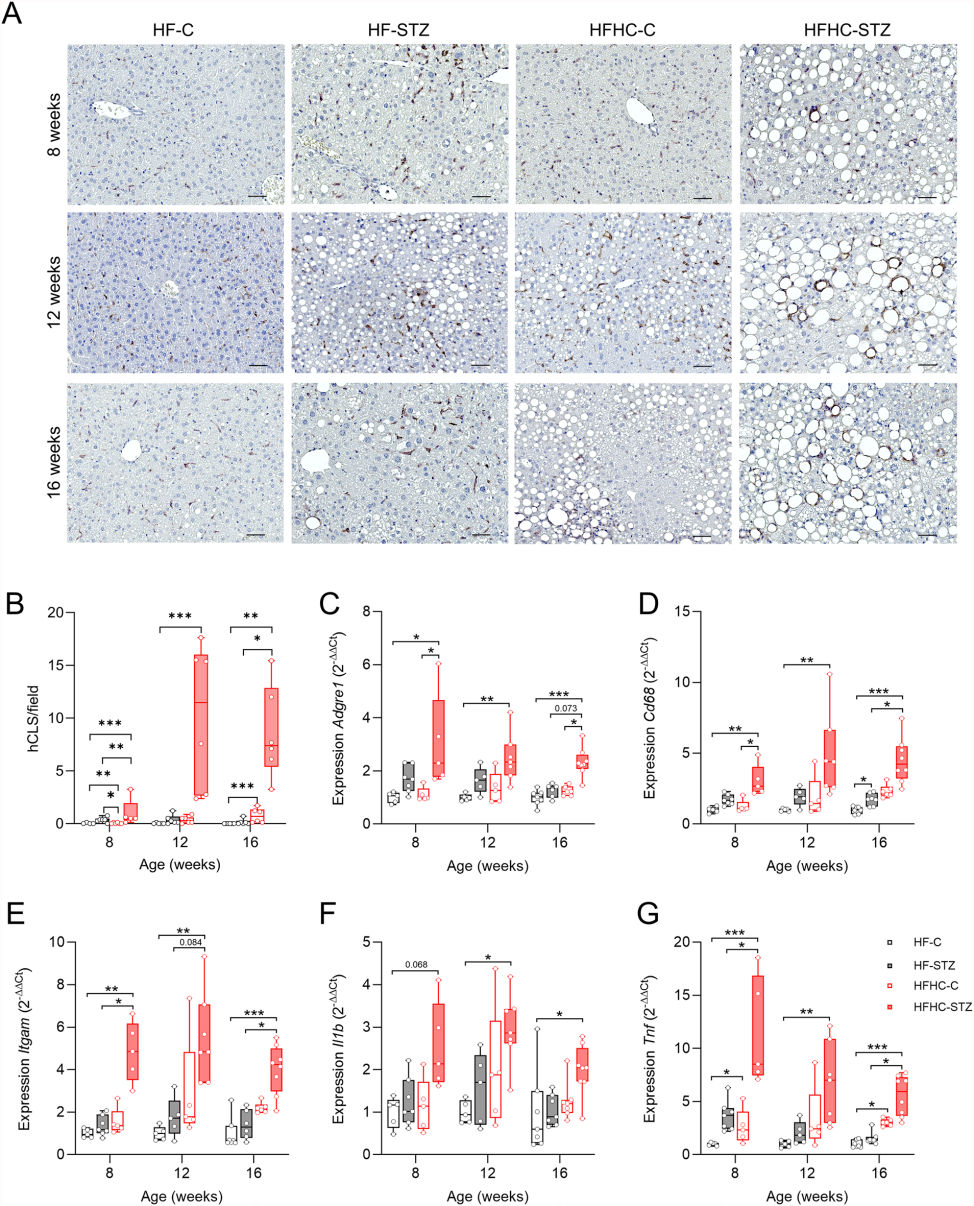
Cholesterol-enriched high-fat diet exacerbates hepatic inflammation in STZ-treated mice. (**A**) Detection and (**B**) quantification of hepatic crown-like structure (hCLS) by immunostaining of macrophage marker F4/80 (*n* = 4–6/group) (scale bars, 50 μm). Hepatic gene expression of macrophage surface proteins (**C**) *Adgre1* (F4/80), (**D**) *Cd68,* and (**E**) *Itgam* (*Cd11b*) and of pro-inflammatory cytokines (**F**) interleukin 1β (*Il1b*) and (**G**) tumor necrosis factor α (*Tnf*). Values are median (line), upper- and lower quartile (box) and extremes (whiskers) (*n* = 5–8/group). Data are analyzed by Kruskal-Wallis test for independent time points. *p < 0.05; **p < 0.01; ***p < 0.001.

To assess hepatic inflammation in our model, we first performed F4/80 immunostaining and quantified hepatic crown-like structures (hCLS), defined as aggregates of immune cells surrounding dying or dead hepatocytes that contain large lipid droplets^33^. hCLS were more abundant in HFHC-STZ animals compared to all other groups (Fig. 4A, B). This histological finding of enhanced inflammation, along with higher score for lobular inflammation in the liver under HFHC-feeding (Fig. 2C and Fig. S2B, E), was supported by the expression of common macrophage surface proteins and pro-inflammatory cytokines. Already at 8 weeks of age, and consistently at later time points, expression of the macrophage marker *Adgre1* (adhesion G protein-coupled receptor E, encoding the protein F4/80) was significantly elevated in HFHC-STZ compared to control animals on both diets (Fig. 4C). Similar trends were observed for *Cd68* (CD68 antigen, Fig. 4D) and *Itgam* (integrin subunit alpha M, encoding the protein CD11b, Fig. 4E). Furthermore, the major inflammatory mediators of macrophages *Il1b* (interleukin 1-beta, Fig. 4F*)* and *Tnf* (tumor necrosis factor α, Fig. 4G) were significantly increased in HFHC-STZ mice compared to the other groups. Notably, the expression of *Tnf* decreased over time in HFHC-STZ mice, whereas it remained relatively stable in all other groups.

**Fig. 5:**
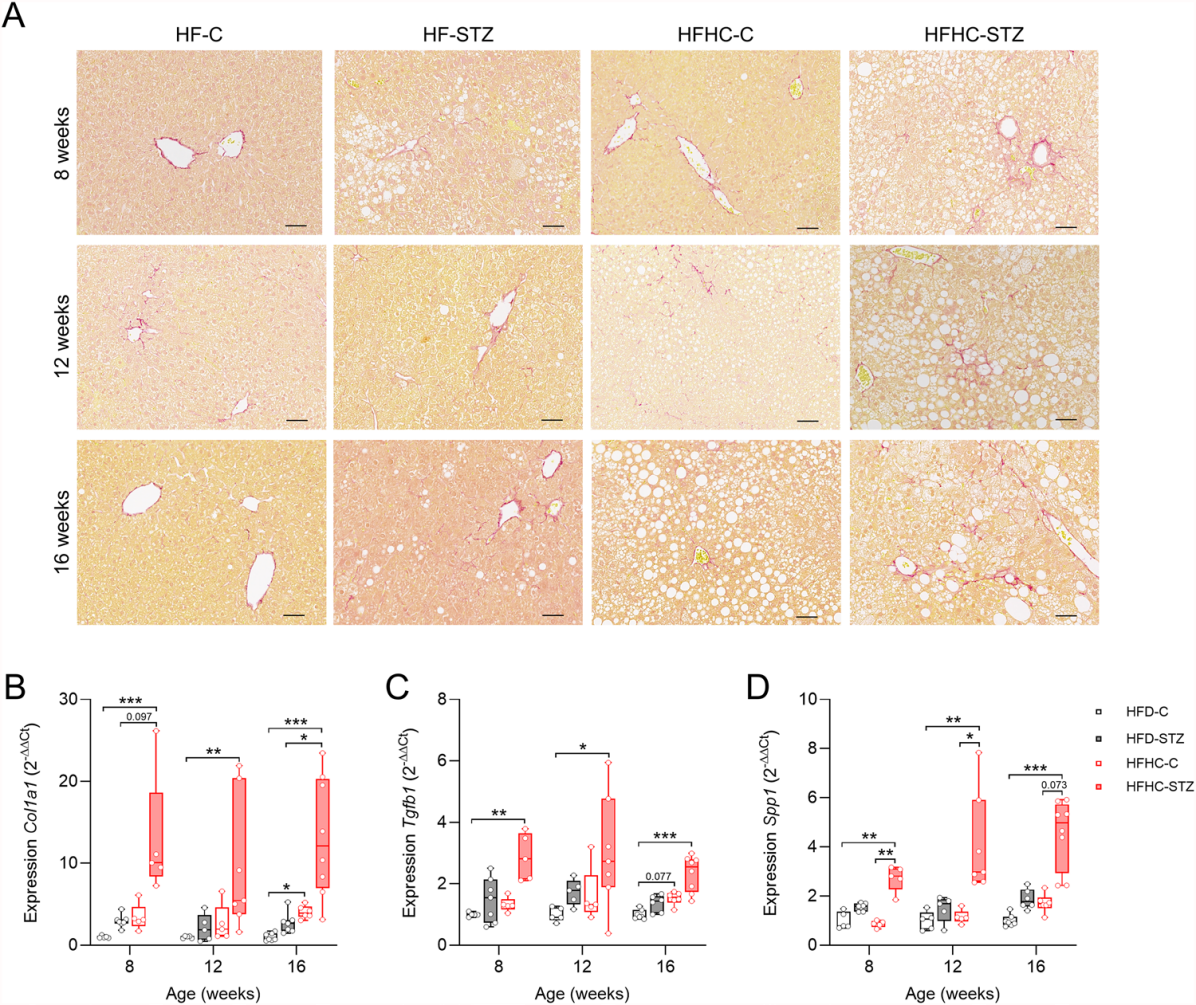
Cholesterol-enriched high-fat diet elevates hepatic fibrosis in STZ-treated mice. (**A**) Representative Sirius red staining of liver sections from mice at 8, 12 or 16 weeks of age (scale bars, 50 μm). Gene expression of (**B**) collagen type 1 (*Col1a1*), (**C**) transforming growth factor β1 (*Tgfb1*), and (**D**) osteopontin (*Spp1*). Values are median (line), upper- and lower quartile (box) and extremes (whiskers) (*n* = 5–8/group). Data are analyzed by Kruskal-Wallis test for independent time points. *p < 0.05; **p < 0.01 ***p < 0.001.

We then examined the effect of STZ treatment in combination with an HFHC diet on the development of fibrosis. Overall, collagen deposition, as indicated by Sirius Red staining of sections, was most pronounced in HFHC-STZ animals and increased over time, particularly in areas containing hCLS (Fig. 5A). Semi-quantitative fibrosis staging confirmed these histological observations (Fig. 2E and Fig. S2C, F). Consistent with the histology, expression of fibrosis-related genes was elevated, including *Col1a1* (collagen type I), *Tgfb1* (transforming growth factor-β), and *Spp1* (secreted phosphoprotein 1, encoding osteopontin protein) in the livers of HFHC-STZ animals (Fig. 5B-D).

To gain insight into the different molecular mechanisms that determine the liver pathology elicited by the two different diets, we performed whole-genome RNA sequencing analysis on the livers of mice at 8 and 12 weeks of age, which define the stages of MASH and fibrosis, respectively, according to the STAM model.

**Fig. 6:**
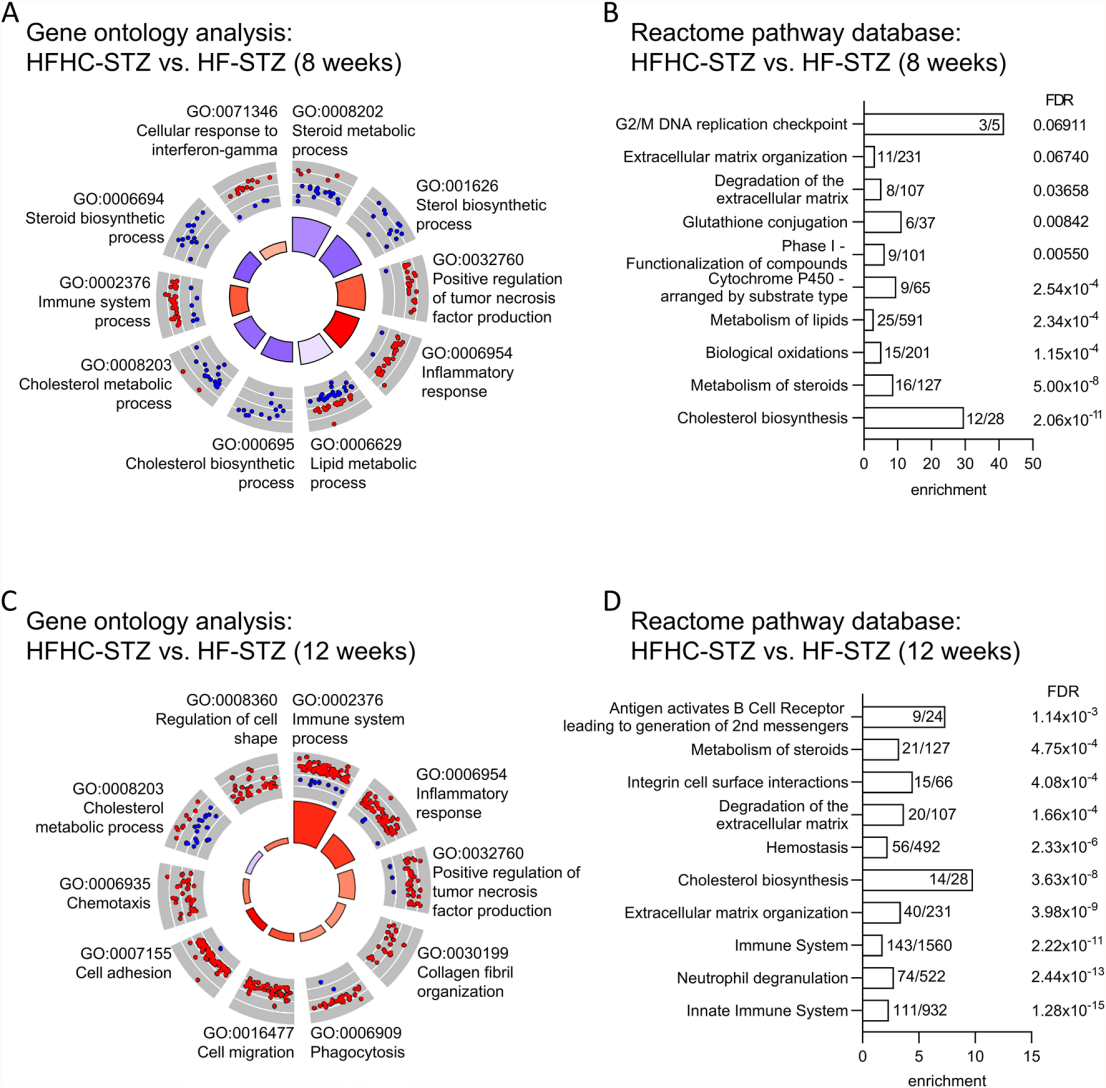
Liver transcriptome changes in STZ-treated mice fed high-fat or cholesterol-enriched high-fat diet. Gene enrichment analysis of differentially expressed genes at 8 and 12 weeks of age (*n* = 4–5/group). (**A**, **C**) Gene ontology (GO) analysis the inner circle depicts the main processes to be increased (red) or decreased (blue). The outer circle shows scaled scatter plots for affected genes and their regulation within the most-enriched biological pathways in HFHC-fed mice. (**B**, **D**) Enrichment of genes in Reactome pathway database including the number of genes allocated to the pathways.

Comparison of STZ-treated mice indicated that dietary cholesterol enrichment further enhanced inflammatory and fibrotic processes in this model. At 8 weeks of age, 256 genes were differentially expressed between the two diets. Gene ontology (GO) analysis revealed significant enrichment in genes implicated in steroid (GO:0008202, GO:0006694), lipid (GO:0006629), and cholesterol (GO:0006695, GO:0008203) metabolism, which were generally downregulated in HFHC-STZ compared to HF-STZ. In contrast, inflammatory (GO:0006954) as well as immune system (GO:0002376) response were upregulated in these mice (Fig. 6A). At the later age, 771 genes were differentially expressed and could be allocated to immune system process (GO:0002376) and inflammation-related (GO:0006954, GO:0032760) GO annotations (Fig. 6C). Enrichment of genes for collagen fibril organization (GO:0030199) can be linked to increased hepatic fibrosis in this age group. Furthermore, pathways related to cell migration (GO:0016477) and chemotaxis (GO:0006935) were induced. Similar terms were identified in the Reactome database (Fig. 6B, D). Moreover, signaling pathways related to extracellular matrix remodeling (organization and degradation) were significantly affected at both time points. The visualization of significant gene sets (FDR < 0.05) in interaction networks also supports the progression of the pathology, with enrichment of inflammation-related pathways from 8 to 12 weeks (Fig. S5). In support of our findings, targeted analysis of MASH/fibrosis-associated gene sets^34^, focusing on bile acid metabolism, ECM organization, hepatocellular death, and inflammation, corroborated these findings (Fig. S6).

Collectively, these data demonstrate that cholesterol enrichment within an HFHC diet substantially exacerbates the pathological progression of fatty liver disease in the STAM model, amplifying inflammatory and fibrotic responses beyond those induced by a high-fat diet alone.

## Discussion

The etiology of human MASLD is highly diverse, comprising a range of genetic risk factors and a multitude of metabolic, nutritional, and environmental contributors^35,36^. Given this complexity, it is unlikely that any single mouse model can fully represent the intricacies of human MASH pathology. However, models can capture certain aspect of the human disease which are useful for developing and testing of therapeutics. Diet composition is particularly crucial in this context. Not only the fat proportion, but also the source of fat and the cholesterol content are decisive for disease features^16^. Whereby the combination of both, a high fat and cholesterol content is especially important^23^. We have shown that a HFHC diet enriched with 0.75% cholesterol and containing soybean oil-derived ω6-poly-unsaturated fatty acids (ω6-PUFA) is superior in driving inflammation and fibrosis compared to lard-based HFHC^22^.

Although 0.75% cholesterol exceeds typical human intake of ∼0.02%, cholesterol handling in mice, including absorption, differs from that of humans, blunting hepatic accumulation^37^. To address this, many models add cholic acid to enhance cholesterol absorption. However, because cholic acid can independently cause liver injury and alter bile acid metabolism^38^, in the present study we instead used 0.75% dietary cholesterol to achieve hepatic cholesterol loading^21^. Consistent with this design, mice on HFHC had higher total hepatic cholesterol than HF-fed mice and exhibited graded, time-dependent increases in NASH-CRN steatosis, lobular inflammation, ballooning, and fibrosis. Thus, our model targets cholesterol-burden–driven injury rather than attempting to replicate average intake or an elevated-synthesis phenotype per se.

Our results do not align with MASH models of insulin resistance which are characterized by hyperinsulinemia and adipose tissue inflammation^34,39^. However, due to the wasting of adipose tissue and hypoinsulinemia, we have used a valuable model for late-stage T2D to test the effect of cholesterol on the pathology. The findings of this study imply that the utilization of HFHC diet in the established STAM model, which has been shown to induce MASH and HCC feeding a lard-based HF diet^17^, results in the exacerbation of pathological features, including steatosis, inflammation, and fibrosis. Genome-wide expression data indicate that HFHC diet affects biosynthesis and metabolism of cholesterol, lipids and steroids in STZ-treated animals at 8 weeks of age (Fig. 6A, B and Fig. S5A). Further progression of the pathology is evident by a massive activation of inflammatory pathways along with a remodeling of the extracellular matrix (ECM) (Fig. 6C, D) which both result in the observed inflammation and fibrosis.

The progression from simple steatosis to MASH in the liver depends among other mechanisms on the activation of tissue-resident Kupffer cells and the transformation of hepatic stellate cells to collagen-producing myofibroblasts. Both processes can be potentiated by hepatic cholesterol burden^40^. The utilized HFHC diet has already proven in a C57BL/6J model of NASH to elevate free cholesterol in comparison to a lard-derived HF diet^21^. In the current study, the expression data also suggest a shift toward hepatic cholesterol accumulation, reflected by lower expression of bile acid synthesis gene (*Cyp7a1*) and by expression pattern which would be compatible with diminished cholesterol esterification (lower *Acat2*, Fig. S4A); because we did not directly quantify free versus esterified cholesterol, we interpret these changes cautiously. Despite elevated total cholesterol levels in the livers of HFHC mice, we observed increased expression of the ABC transporters ABCG5/G8 (*Abcg5* and *Abcg8*), which normally facilitate the efflux of cholesterol. This finding is in contrast to results reported earlier in mice and in human MASH^21,41^, but aligns to expression pattern in another preclinical model^23^.

There is increasing evidence of the toxicity of free cholesterol in the liver, which can precipitate and form crystals that promote the development of MASH^25,42^. Although we did not measure free cholesterol or crystals, we overserved increased aggregation of macrophages around lipid-loaded dying hepatocytes in so called hepatic crown-like structures in liver of HFHC-STZ mice (Fig. 4A, B). It appears conceivable that the elimination of dead hepatocytes leads to the uptake of cholesterol crystals by macrophages, thereby activating the NLRP3 inflammasome. This, in turn, potentiates an inflammatory response that includes the secretion of fibrogenic cytokines such as TNFα and TGFβ^28,43^, both induced by HFHC (Figs. 4G and 5C). However, experimental models have also demonstrated that Kupffer cells and the inflammatory process can be directly activated by cholesterol. Scavenger-receptor-mediated uptake of modified LDL can lead to lysosomal accumulation of cholesterol^44^, which consequently triggers the inflammation^45^.

In addition to the induction of inflammation, we observed more severe fibrosis in HFHC-STZ animals, as indicated by collagen deposition and elevated expression of collagen type 1 (*Col1a1*). Furthermore, the expression of *Spp1*, which encodes osteopontin, has been positively associated with the stage of fibrosis in human MASH and mouse models and was also elevated in the liver of HFHC-STZ mice (Fig. 5B, D)^46–48^. Furthermore, implementation of the Reactome pathway database analysis revealed enrichment of genes in ECM-remodeling (Fig. 6D). In the liver, fibrosis is mediated through transdifferentiation of hepatic stellate cells from a quiescent state into myofibroblast^49^. These cells are the main source of ECM in fibrosis development^50^. The transformation of stellate cells is induced by TNFα and TGFβ, which are primarily secreted by activated Kupffer cells. Consequently, stellate cell transformation is associated with free cholesterol and cholesterol crystals.

## Limitations

Human MASH most commonly arises in the context of obesity, insulin resistance, and hyperinsulinemia. The STAM model used here reproduces steatosis, inflammation, and fibrosis but more closely reflects late-stage T2D with hyperglycemia (Fig. 1B) and loss of adipose mass (Fig. S1), without hyperinsulinemia (Fig. 1A) typical present in insulin-resistant states. Additionally, while our HFHC diet increased total hepatic cholesterol and exacerbated NASH-CRN endpoints, high dietary cholesterol suppresses SREBP2/HMGCR in mice^23^, whereas many patients with MASH exhibit increased cholesterol synthesis^40^. Accordingly, our study was designed to model the consequences of hepatic cholesterol burden in the STAM model, not the synthesis phenotype, and conclusions about synthesis-targeted mechanisms should be drawn cautiously. We did not quantify free versus esterified hepatic cholesterol or visualize crystals; future work should address these endpoints (e.g., enzymatic assays, filipin staining, and polarization microscopy) and further delineate the contributions of bile acid metabolism.

## Methods

### Animals and experimental design

MASH was induced in male C57BL/6J mice as reported previously^17^. At the age of two days, mice were injected with single subcutaneous dose of 200 µg streptocotozin (STZ; Sigma-Aldrich, Darmstadt, Germany), dissolved in 0.1 M citrate buffer (pH 4.5). Control mice (C) received the solvent solution. At 4 weeks of age mice were switched to *ad libitum* feeding of a high-fat diet containing lard as fat source (HF; E15742-34; 60 kcal% fat, 20 kcal% carbohydrates and 20 kcal% protein; Ssniff, Soest, Germany) or a custom-made cholesterol-enriched high-fat diet containing ω6-PUFA-rich soybean oil (HFHC; 49 kcal% fat, 35 kcal% carbohydrates and 16 kcal% protein, 0.75% cholesterol; Altromin, Lage, Germany^21)^ for up to 16 weeks of age. Mice were sacrificed after a 6-hour fast at 6, 8, 12 or 16 weeks of age according to disease stages defined by the original description of the STAM model^17^. At the end of the study, the mice were placed under deep isoflurane anesthesia. After confirming their lack of responsiveness to a toe pinch, the mice were euthanized by cardiac blood draw followed by cervical dislocation. Plasma and organs were snap-frozen in liquid nitrogen and stored at -80 °C for biochemical analysis; organs were fixed for histological examination.

Animal experiments were performed referring to the ARRIVE guidelines. All experiments were approved by the ethics committee of the State Agency of Environment, Health, and Consumer Protection (State of Brandenburg, Germany, 2347-33-2021). We confirm that all experimental procedures were conducted in accordance with the above regulations and guidelines.

### Body composition and in vivo experiments

Body composition was determined by non-invasive nuclear magnetic resonance spectroscopy (EchoMRI™-100 system, Echo Medical Systems, Houston, TX, USA). Blood glucose was measured in the morning with a CONTOUR^®^ XT glucometer (Bayer Consumer Care AG, Leverkusen, Germany).

### Plasma analysis

Triglyceride, glycerol (TR0100, Sigma-Aldrich) and NEFA (NEFA-HR(2), Wako Chemicals GmbH, Neuss, Germany) were measured in plasma according to manufacturers’ instruction. Total plasma cholesterol was measured using the Cholesterol liquicolor kit (Human Diagnostics Worldwide, Wiesbaden, Germany). Insulin levels in plasma were assessed using a respective ELISA (80-INSMSU-E01, ALPCO Diagnostics, Salem, NH, USA) following manufacturer’s protocol. Aspartate aminotransferase (AST) and alanine aminotransferase (ALT) activity were spectrophotometrically assessed in blood plasma using commercially available kits (Human Diagnostics Worldwide). Absorbance was measured at 340 nm by using a SpectraMax (Molecular Devices, San José, CA, USA).

### Detection of liver triglycerides and cholesterol

Liver triglycerides (RandoxTR-210, Crumlin, UK) and cholesterol levels (10017, Human Diagnostics Worldwide) were quantified enzymatically using commercially available kits. In brief, liver tissue were homogenized for 5 min in 10 mmol/L sodium dihydrogen phosphate, 1 mmol/L EDTA, and 1% (vol./vol.) polyoxyethylene-10-tridecyl ether and incubated for 5 min at 70 °C to inactivate enzymes, kept on ice for 5 min, and centrifuged. If a visible lipid layer formed, it was reincorporated by re-homogenization and dilution with homogenization buffer containing detergent, followed by re-centrifugation; this was repeated until a clear, single-phase supernatant was obtained. The final clear supernatant, which contained the solubilized lipids, was used for the enzymatic assays. Values were normalized to tissue weight.

### Histological stainings

Liver tissue was fixed in 4% w/v paraformaldehyde for 24 h at 4 °C and dehydrated by increasing concentrations of ethanol and xylene. Tissue was embedded in paraffin wax, sectioned into 5 μm slices and stained with hematoxylin and eosin (H&E). To assess fibrosis, liver sections were stained with Sirius red (SR). The histopathological scoring was performed on H&E- and SR-stained liver sections by an experienced histopathologist blinded to experimental group assignment using the NAFLD Activity Score (NAS) and fibrosis staging^51^. The NAS ranging from 0 to 8 was calculated as the sum of the individual scores for steatosis (0–3), lobular inflammation (0–3), and hepatocellular ballooning (0–2). Liver fibrosis was scored based on a five-point scale, i.e. absence of fibrosis (stage 0), perisinusoidal or portal fibrosis (stage 1), perisinusoidal and portal/periportal fibrosis (stage 2), septal or bridging fibrosis (stage 3), cirrhosis (stage 4). For quantification of hepatic crown-like structures (hCLS), liver sections were immunostained with F4/80 antibody (ab6640, Abcam, Cambridge, UK). Antigen-antibody complexes were detected using a biotinylated rabbit anti-rat secondary antibody (Dako, Glostrup, Denmark) and the VECTASTAIN Elite ABC-HRP system (Vector Laboratories, Newark, CA, USA), visualized with 3,3′-diaminobenzidine (DAB, Dako), and counterstained with hematoxylin. Microscopy was performed with the Axio Scan.Z1 slide scanner (Zeiss, Jena, Germany).

### Gene expression analysis

Total RNA of liver tissue was isolated using RNeasy Mini Kits (Qiagen, Hilden, Germany), and cDNA was prepared by M-MLV Reverse Transcriptase-Kit (Promega, Madison, WI, USA). Hydrolysis probes (Table 1, Integrated DNA Technologies, Leuven, Belgium) were applied for quantitative reverse transcription PCR (qRT-PCR). *Eef2* (eukaryotic translation elongation factor) and *Ppia* (Peptidylpropyl isomerase A) were used as reference genes. Relative gene expression was analyzed using the ΔΔCt method.

**Table 1:** Hydrolysis probes for qRT-PCR.

Gene symbol	Gen name	Assay-ID
*Adgre1*	Adhesion G protein-coupled receptor E1	Mm.PT.58.11087779
*Cd68*	CD68 antigene	Mm.PT.58.8802827
*Col1a1*	Collagen, type I, alpha 1	Mm.PT.58.7562513
*Eef2*	Eukaryotic translation elongation factor	Self designed*
*Il1b*	Interleukine 1 beta	Mm.PT.58.41616450
*Itgam (Cd11b)*	integrin alpha M	Mm.PT.58.14195622
*Ppia*	Peptidylpropyl isomerase A	Mm.PT.39a.2.gs
*Spp1*	Secreted phosphoprotein 1	Mm.PT.58.43709208
*Tgfb1*	Transforming growth factor, beta 1	Mm.PT.58.11254750
*Tnf*	Tumor necrosis factor	Mm.PT.58.12575861

* Probe: 5´-TAAGCAGAG-[ZEN]-CAAGGATGGCT-3´

Primer1: 5´-TGAGGTTGATGAGGAAGCCC-3´

Primer2: 5´-CACAATCAAATCCACCGCCA-3´

### RNA sequencing analysis

RNAseq (*n* = 4–5 mice/ group) was performed by BGI (Hong Kong, China). To apply high-quality RNA for RNAseq analysis we assessed RNA integrity by using a Bioanalyzer and an appropriate kit (RNA6000 nano kit; Agilent, Santa Clara, CA, USA). All preparations were made according to manufacturer’s recommendations. Only RNA with a minimal RNA integrity number of 8.0 (RIN) was used. Sequencing was performed with DNBSeq-G400, generating paired end reads with 150 bp length, and at least 36*10^6^ cleaned reads per sample. Significant genes were counted as adjusted *p*-value < 0.05 and an absolute Log2FC > = 1. The raw data has been uploaded to GEO and can be found with the ID GSE319011. The R package DESeq2 was used for differential gene expression analysis. *P*-values values were adjusted using the Benjamini-Hochberg Method, values < 0.05 were considered as significant. Gene ontology (GO) analysis (biological process) and Reactome was performed using Database for Annotation, Visualization and Integrated Discovery (DAVID). GO networks were visualized using Cytoscape-3.10.2 and EnrichmentMap 3.5.0 plug-in^52,53^.

### Statistical analysis

Values are presented as median (line), including upper and lower quartile (box) and extremes (whiskers) or as means ± SEM. Statistical analysis was performed by two-way ANOVA (mixed model) with Tukey’s multiple comparisons test or Kruskal-Wallis test. Differences were considered significant when *p* < 0.05. Statistical analysis was conducted by GraphPad Prism 10 (GraphPad Software Inc., La Jolla, CA, USA).

## Supplementary Information

Below is the link to the electronic supplementary material.

Supplementary Material 1

## Data Availability

Our sequencing data were submitted to the Gene Expression Omnibus (GEO; http://www.ncbi.nlm.nih.gov/geo/) repository GSE319011. Other relevant data are available from the corresponding authors upon reasonable request.
