# Supplementary material for: Cholesterol-rich diet exacerbates steatohepatitis in the STAM mouse model
Source: Sci Rep. 2026 Apr 1;16:11231. doi: 10.1038/s41598-026-45979-z (PMC13046726; doi:10.1038/s41598-026-45979-z)
Supplement: Supplementary file 1 — Supplementary Material 1 [file 41598_2026_45979_MOESM1_ESM.pdf]

Supplementary Material

Cholesterol-rich diet exacerbates steatohepatitis in the STAM mouse model

Wenke Jonas, Pascal Gottmann, Markus Jähnert, Nora Baer, Annette Schürmann and Heike Vogel

Supplementary table

Table S1. Plasma parameters

Data are presented as mean±SEM and are analyzed by Kruskal-Wallis test and Tukey's multiple comparison test. Different letters indicate statistically significant differences. Values for transaminases ALT and AST were log transformed, tested for normal distribution and analyzed by parametric One-way ANOVA (*n* = 5-8/per group). Abbreviations: C, control; HF, high-fat diet; HFHC, cholesterol-enriched high-fat diet; STZ, streptocotozin.

	HF-C	HF-STZ	HFHC-C	HFHC-STZ
	**8 weeks**			
ALT (U/L)	28.2 ± 2.1[a]	55.1 ± 8.0[bc]	33.3 ± 8.8[ab]	75.7 ± 20.3[c]
AST (U/L)	39.7 ± 3.6[a]	80.3 ± 6.2[b]	35.0 ± 4.6[a]	78.57 ± 15.4[b]
FFA (mM)	0.42 ± 0.03[a]	0.63 ± 0.10[a]	0.50 ± 0.03[a]	0.51 ± 0.04[a]
Glycerol (µg/mL)	297.2 ± 25.8[a]	336.4 ± 33.1[a]	388.6 ± 36.6[a]	298.9 ± 22.1[a]
	**12 weeks**			
ALT (U/L)	29.9 ± 8.1[a]	39.7 ± 4.9[ab]	41.8 ± 7.5[ab]	123.6 ± 42.3[b]
AST (U/L)	49.6 ± 6.8[a]	74.8 ± 6.2[ab]	51.2 ± 5.8[a]	160.4 ± 54.3[b]
FFA (mM)	0.63 ± 0.07[a]	0.62 ± 0.08[a]	0.54 ± 0.03[a]	0.56 ± 0.06[a]
Glycerol (µg/mL)	429.3 ± 27.5[a]	376.7 ± 40.2[ab]	288.0 ± 19.1[b]	404.5 ± 100.8[ab]
	**16 weeks**			
ALT (U/L)	31.9 ± 4.6[a]	67.9 ± 15.5[ab]	92.1 ± 34.4[ab]	174.3 ± 60.1[b]
AST (U/L)	53.7 ± 6.8[a]	84.8 ± 15.1[ab]	76.0 ± 18.8[ab]	174.4 ± 46.6[b]
FFA (mM)	0.53 ± 0.03[a]	0.40 ± 0.06[a]	0.46 ± 0.06[a]	0.60 ± 0.08[a]
Glycerol (µg/mL)	391.3 ± 38.3[a]	296.4 ± 41.4[a]	355.7 ± 25.8[a]	384.3 ± 20.8[a]

Supplementary figures

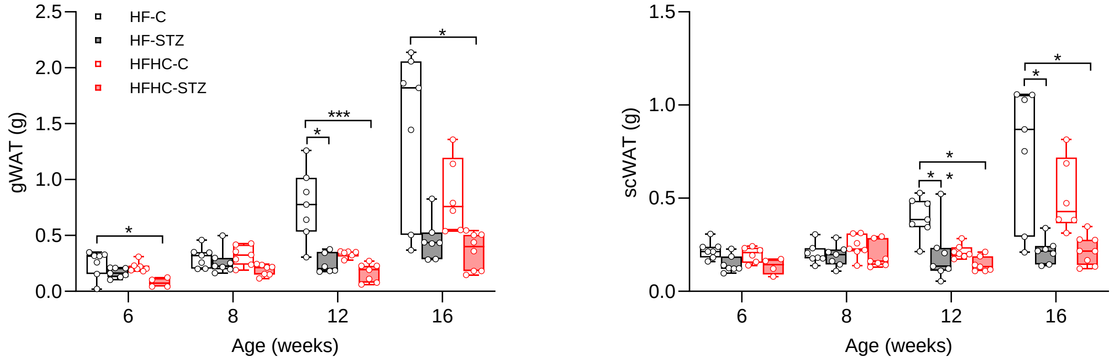

Fig. S1. Fat depots of STZ-treated mice are smaller. Weight of **(A)** gonadal white adipose tissue (gWAT) depot and **(B)** subcutaneous white adipose tissue (scWAT) depot. Values are median (line), upper and lower quartile (box) and extremes (whiskers) (*n* = 4-8/group). Data are analyzed by Kruskal-Wallis test for independent time points. *$p < 0.05$; **$p < 0.01$; ***$p < 0.001$.

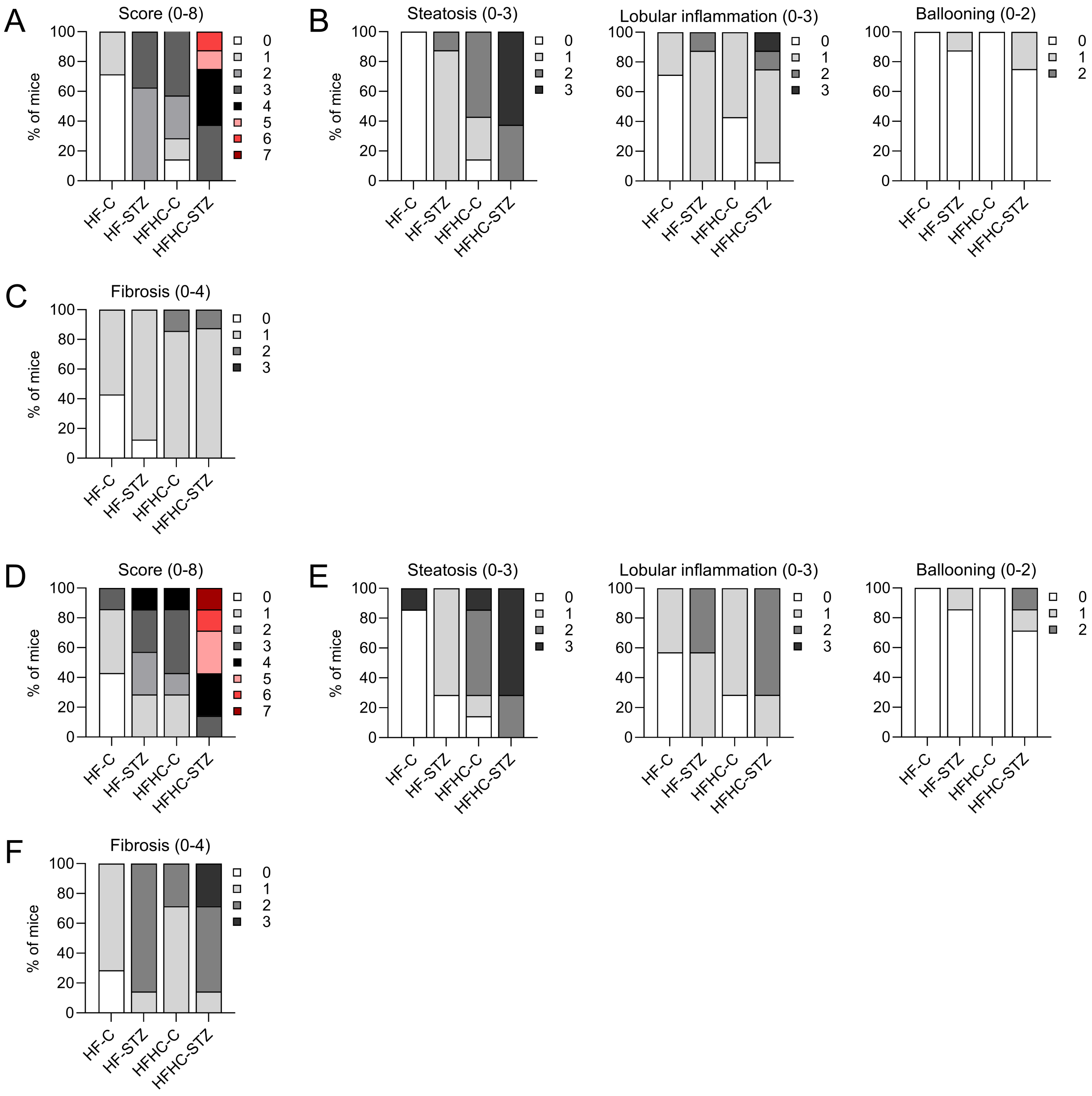

Fig. S2. Histopathological scoring by NAFLD activity score (NAS) and fibrosis stage. NAS calculated as sum for steatosis, lobular inflammation, and hepatocellular ballooning, and scoring of fibrosis stage in **(A-C)** 8- and **(D-F)** 12-weeks old mice (*n* = 4-7/group).

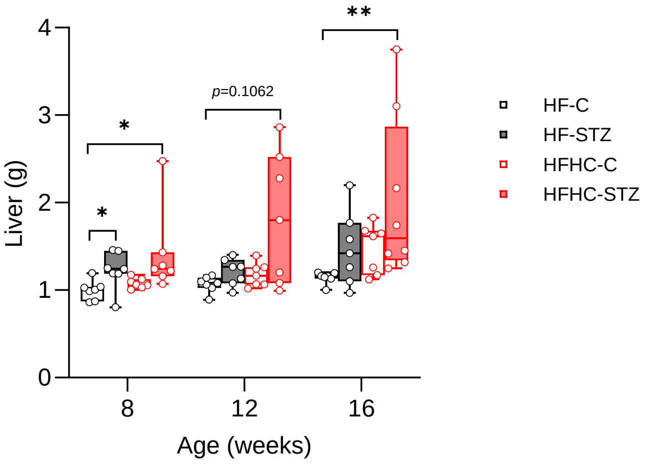

Fig. S3. Absolute liver weight. Values are median (line), upper and lower quartile (box) and extremes (whiskers) (n = 7-8/group). Data are analyzed by Kruskal-Wallis test for independent time points. *$p < 0.05$; **$p < 0.01$.

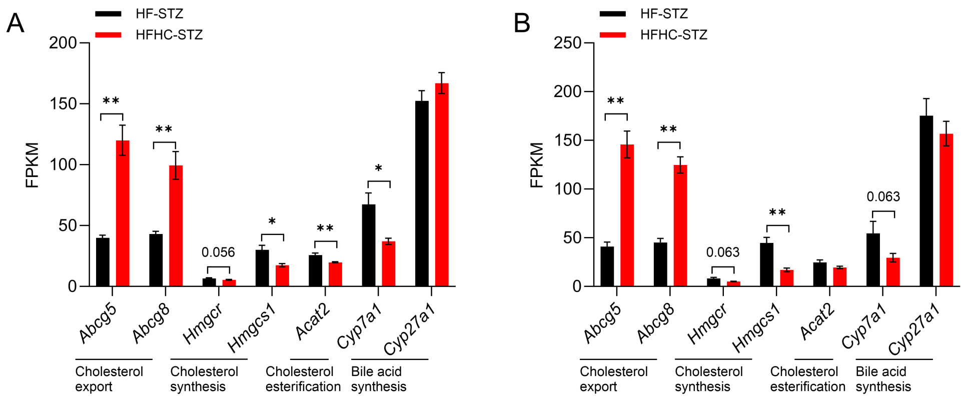

Fig. S4. Expression of genes related to cholesterol metabolism of STZ-treated mice. Hepatic expression data from RNAseq analysis of **(A)** 8- and **(B)** 12-weeks old mice fed with HF or HFHC diet (n = 4-5/group). Cholesterol transporters ATP-binding cassette sub-family G (*Abcg*) 5 and 8, cholesterol esterification enzyme acetyl-CoA acetyltransferase 2 (*Acat2*), cholesterol synthesis enzymes 3-hydroxy-3-methylglutaryl-CoA reductase (*Hmgcr*), 3-hydroxy-3-methylglutaryl-CoA synthase 1 (*Hmgcs1*), cholesterol-metabolizing enzymes cytochrome P450 family 7a1 (*Cyp7a1*), and family 27a1 (*Cyp27a1*). Data are presented as mean±SEM. Data are analyzed by Mann-Whitney test. *$p < 0.05$; **$p < 0.01$.

A

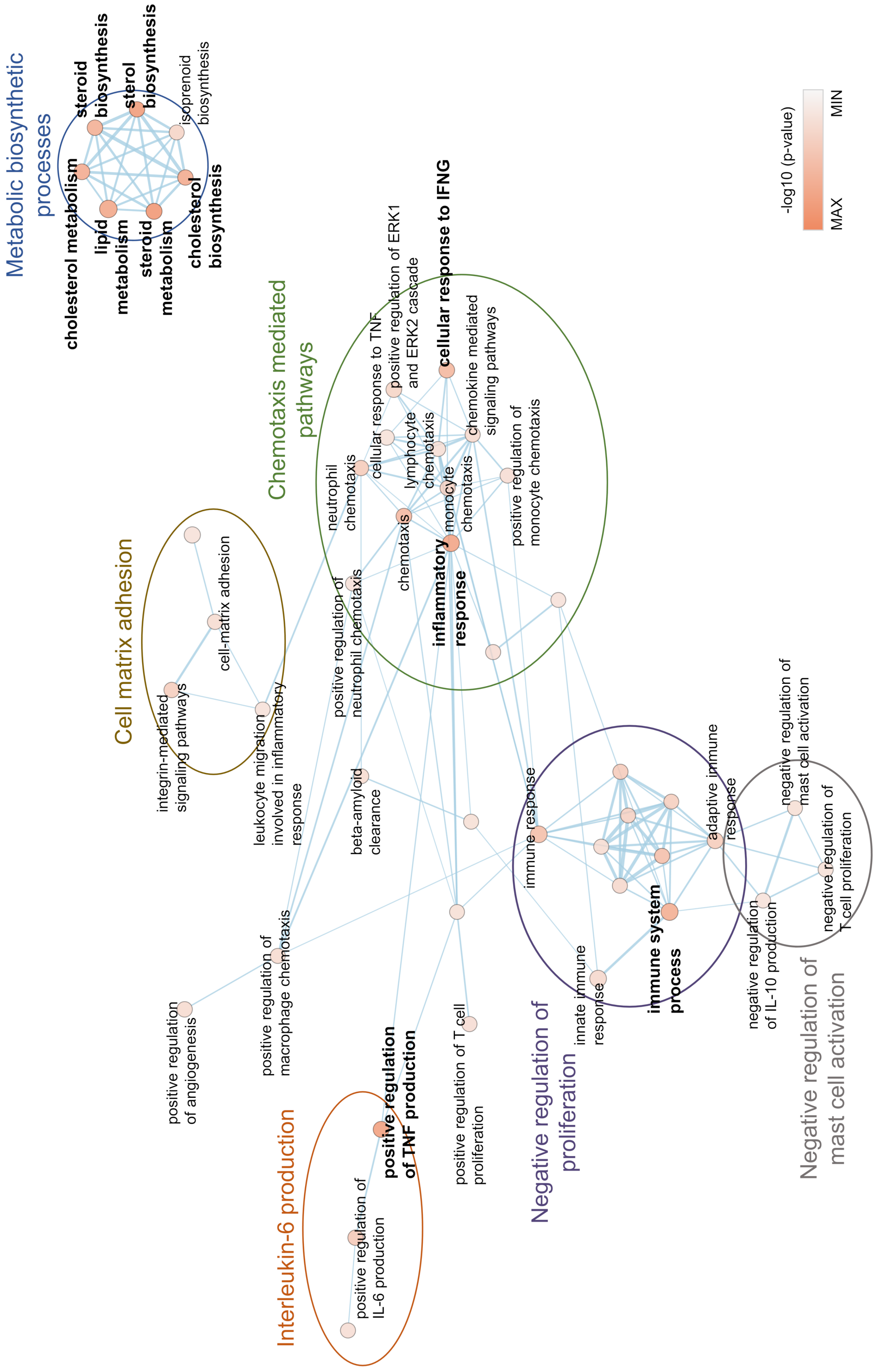
Metabolic biosynthetic processes
steroid biosynthesis
sterol biosynthesis
isoprenoid biosynthesis
cholesterol metabolism
lipid metabolism
steroid metabolism
cholesterol biosynthesis
Chemotaxis mediated pathways
positive regulation of ERK1 and ERK2 cascade
cellular response to IFNG
cellular response to TNF
chemokine mediated signaling pathways
lymphocyte chemotaxis
neutrophil chemotaxis
positive regulation of monocyte chemotaxis
monocyte chemotaxis
chemotaxis
inflammatory response
positive regulation of neutrophil chemotaxis
Cell matrix adhesion
cell-matrix adhesion
integrin-mediated signaling pathways
leukocyte migration involved in inflammatory response
beta-amyloid clearance
positive regulation of macrophage chemotaxis
positive regulation of angiogenesis
positive regulation of TNF production
positive regulation of T cell proliferation
Interleukin-6 production
positive regulation of IL-6 production
Negative regulation of proliferation
immune response
adaptive immune response
innate immune response
immune system process
negative regulation of mast cell activation
negative regulation of T cell proliferation
negative regulation of IL-10 production
Negative regulation of mast cell activation
-log10 (p-value)
MIN
MAX

B

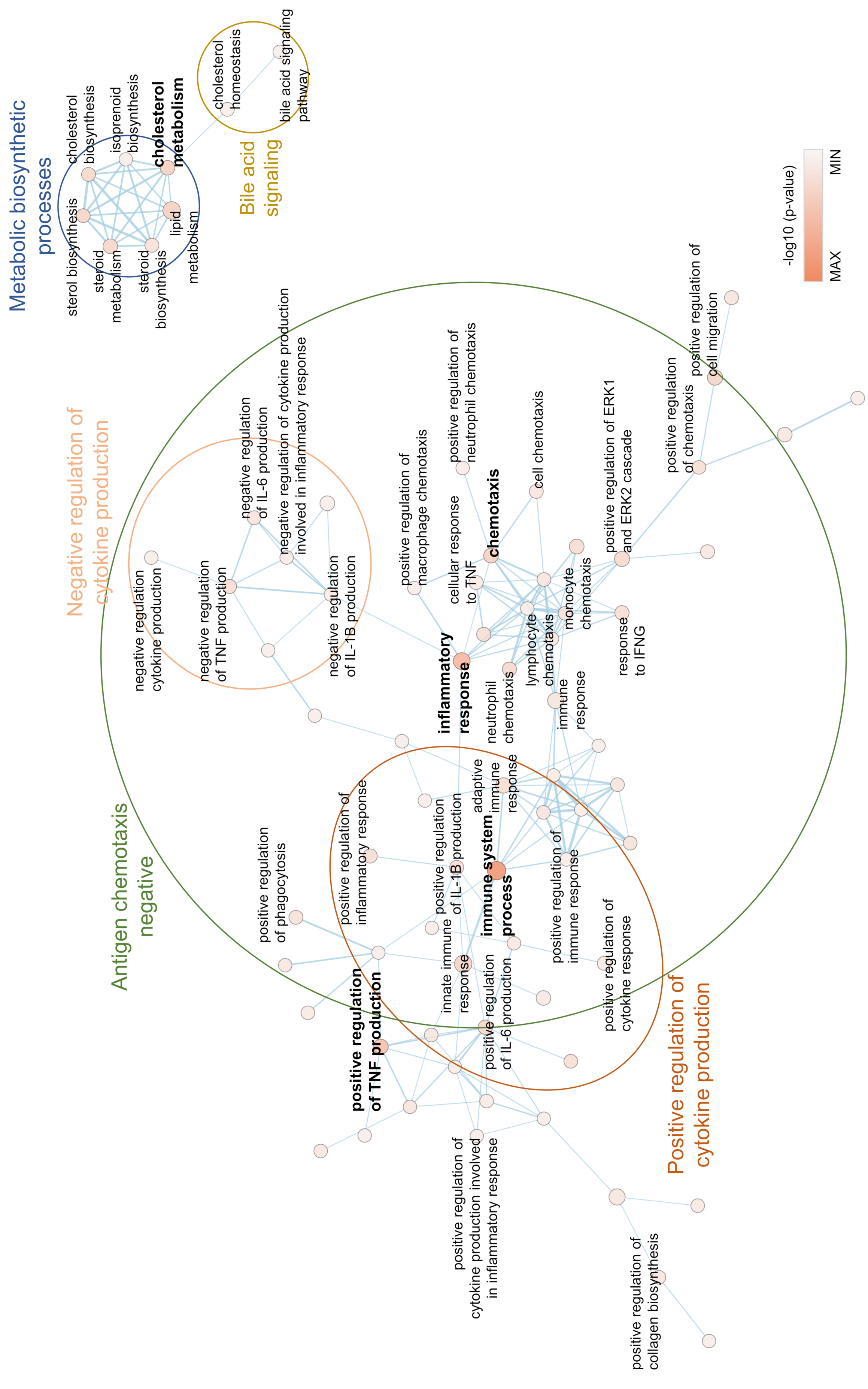

Metabolic biosynthetic processes
cholesterol biosynthesis
isoprenoid biosynthesis
cholesterol metabolism
sterol biosynthesis
steroid metabolism
steroid biosynthesis
lipid metabolism
Bile acid signaling
cholesterol homeostasis
bile acid signaling pathway
-log10 (p-value)
MIN
MAX
Negative regulation of cytokine production
negative regulation cytokine production
negative regulation of TNF production
negative regulation of IL-6 production
negative regulation of cytokine production involved in inflammatory response
negative regulation of IL-1B production
Antigen chemotaxis negative
positive regulation of macrophage chemotaxis
positive regulation of neutrophil chemotaxis
cellular response to TNF
chemotaxis
cell chemotaxis
positive regulation of ERK1 and ERK2 cascade
positive regulation of chemotaxis
positive regulation of cell migration
monocyte chemotaxis
response to IFNG
lymphocyte chemotaxis
inflammatory response
neutrophil chemotaxis
immune response
positive regulation of phagocytosis
positive regulation of inflammatory response
positive regulation of IL-1B production
adaptive immune response
immune system process
innate immune response
positive regulation of immune response
positive regulation of cytokine response
positive regulation of TNF production
positive regulation of IL-6 production
Positive regulation of cytokine production
positive regulation of cytokine production involved in inflammatory response
positive regulation of collagen biosynthesis

Fig. S5. Interaction network of GO terms generated by the Cytoscape plugin EnrichmentMap. Each node represents a GO term. Node color indicates the *p*-value. The networks show DEGs enrichment in livers of mice at **(A)** 8 and **(B)** 12 weeks of age. Top regulated gene ontology (GO) terms (Figure 6A, C) are highlighted in bold. Groups of functionally related gene sets are circled and labelled.

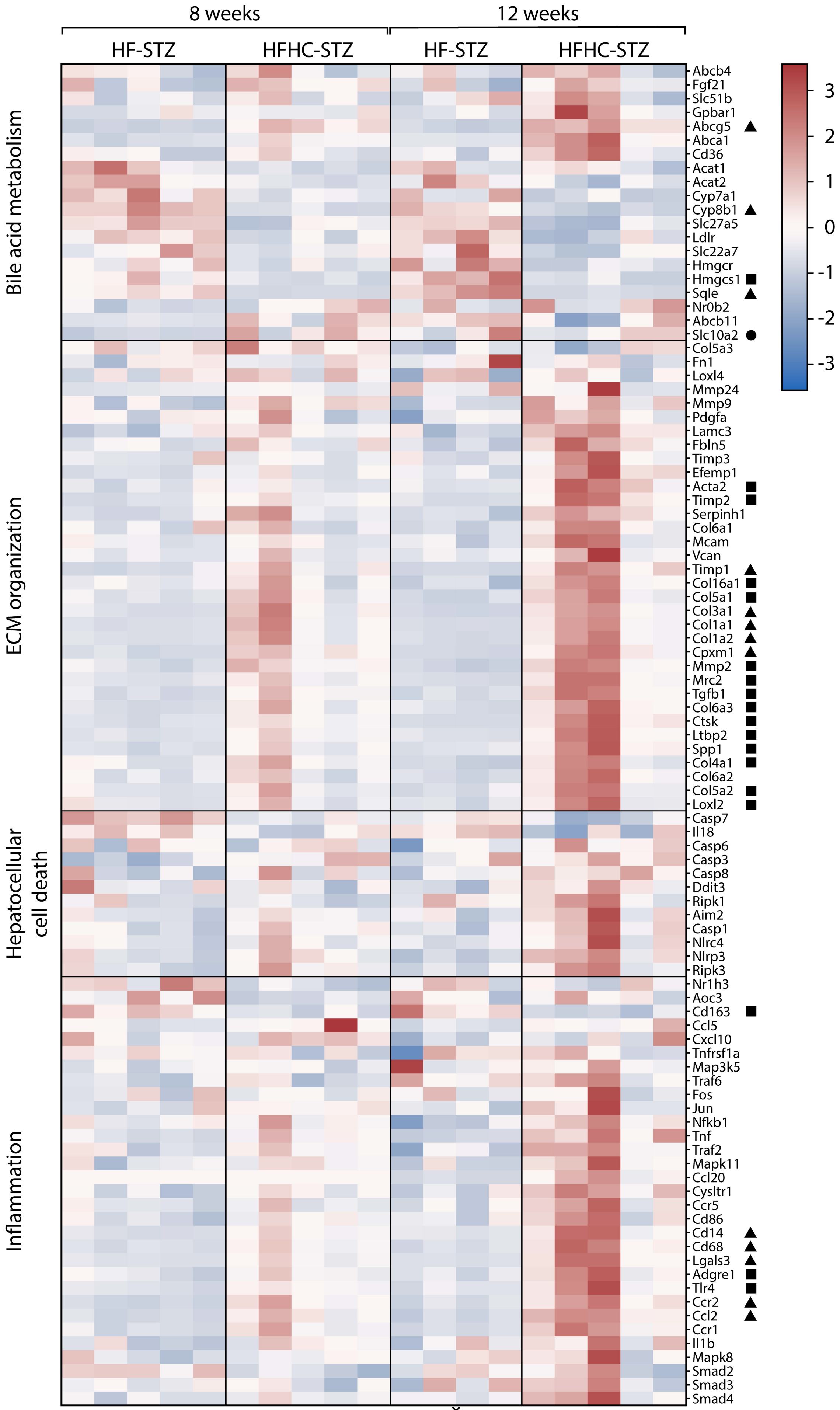
8 weeks
12 weeks
HF-STZ
HFHC-STZ
HF-STZ
HFHC-STZ
Bile acid metabolism
ECM organization
Hepatocellular cell death
Inflammation
Abcb4
Fgf21
Slc51b
Gpbar1
Abcg5
Abca1
Cd36
Acat1
Acat2
Cyp7a1
Cyp8b1
Slc27a5
Ldlr
Slc22a7
Hmgcr
Hmgcs1
Sqle
Nr0b2
Abcb11
Slc10a2
Col5a3
Fn1
Loxl4
Mmp24
Mmp9
Pdgfa
Lamc3
Fbln5
Timp3
Efemp1
Acta2
Timp2
Serpinh1
Col6a1
Mcam
Vcan
Timp1
Col16a1
Col5a1
Col3a1
Col1a1
Col1a2
Cpxm1
Mmp2
Mrc2
Tgfb1
Col6a3
Ctsk
Ltbp2
Spp1
Col4a1
Col6a2
Col5a2
Loxl2
Casp7
Il18
Casp6
Casp3
Casp8
Ddit3
Ripk1
Aim2
Casp1
Nlrc4
Nlrp3
Ripk3
Nr1h3
Aoc3
Cd163
Ccl5
Cxcl10
Tnfrsf1a
Map3k5
Traf6
Fos
Jun
Nfkb1
Tnf
Traf2
Mapk11
Ccl20
Cysltr1
Ccr5
Cd86
Cd14
Cd68
Lgals3
Adgre1
Tlr4
Ccr2
Ccl2
Ccr1
Il1b
Mapk8
Smad2
Smad3
Smad4
3
2
1
0
-1
-2
-3

Fig. S6. Heatmap of disease-associated genes. Heatmap of RNA-seq data expression (z-scores) computed for genes associated with MASH pathology and fibrosis. Each column in the heatmap is an individual sample. Symbols denote significant differences between HFHC-STZ and HF-STZ within each age group: ● week 8, ■ week 12, ▲ week 8 and 12
